# Supplementary figures and images for: Allelic strengths of encephalopathy-associated UBA5 variants correlate between in vivo and in vitro assays
Source: eLife. 2023 Dec 11;12:RP89891. doi: 10.7554/eLife.89891 (PMC10712953; doi:10.7554/eLife.89891)

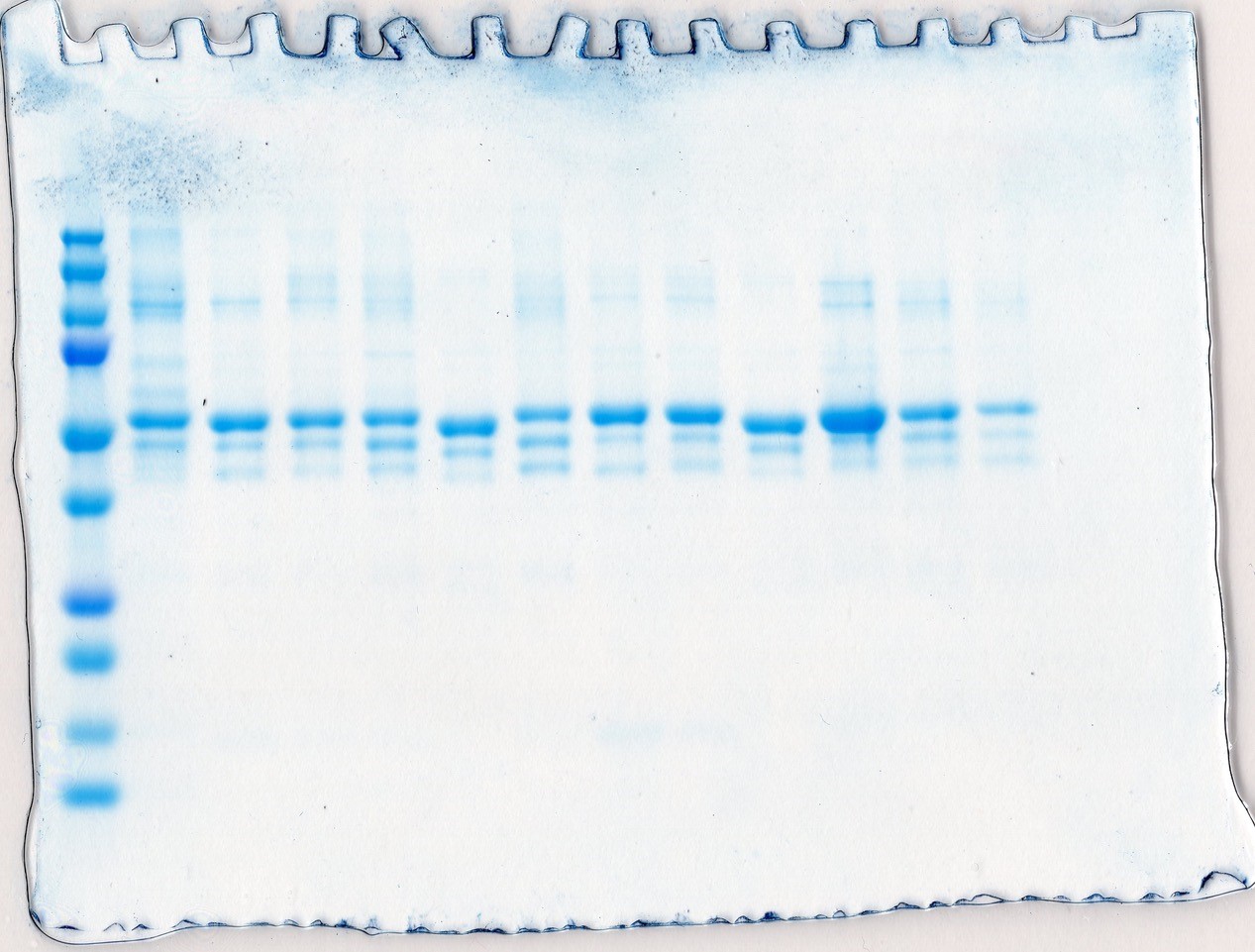

Supplement: Figure 6—source data 1. [file elife-89891-fig6-data1.zip › Fig. 6A.jpg]

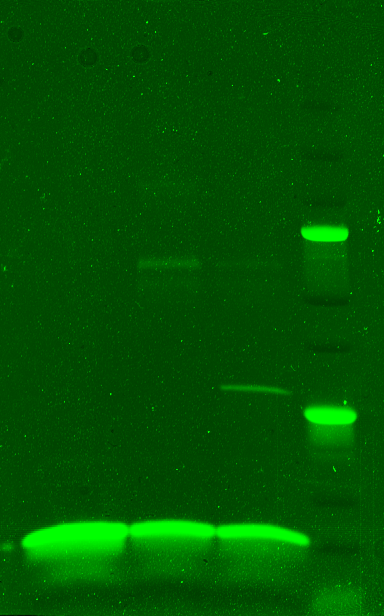

Supplement: Figure 7—source data 1. [file elife-89891-fig7-data1.zip › Fig. 7C.tif]
